# Circulating cell-free DNA and circulating tumor cells, the “liquid biopsies” in ovarian cancer

**DOI:** 10.1186/s13048-017-0369-5

**Published:** 2017-11-13

**Authors:** Xianliang Cheng, Lei Zhang, Yajuan Chen, Chen Qing

**Affiliations:** 1 School of Pharmaceutical Sciences & Yunnan Provincial Key Laboratory of Pharmacology for Natural Products, Kunming Medical University, 1168 West Chun Rong Road, Cheng Gong, Kunming, Yunnan 650500 People’s Republic of China; 2 Department of Gynecologic Oncology, The Third Affiliated Hospital of Kunming Medical University, Kunming, Yunnan People’s Republic of China

**Keywords:** Ovarian cancer, Liquid biopsy, Circulating cell-free DNA, Circulating tumor DNA, Circulating tumor cell, Tumor biomarkers

## Abstract

Limited understanding of ovarian cancer (OC) genome portrait has hindered the therapeutic advances. The serial monitoring of tumor genotypes is becoming increasingly attainable with circulating cell-free DNA (cf-DNA) and circulating tumor cells (CTCs) emerging as “liquid biopsies”. They represent non-invasive biomarkers and are viable, as they can be isolated from human plasma, serum and other body fluids. Molecular characterization of circulating tumor DNA (ct-DNA) and CTCs offer unique potentials to better understand the biology of metastasis and resistance to therapies. The liquid biopsies may also give innovative insights into the process of rapid and accurate identification, resistant genetic alterations and a real time monitoring of treatment responses. In addition, liquid biopsies are shedding light on elucidating signal pathways involved in invasiveness and metastasis competence; but the detection and molecular characterization of ct-DNA and CTCs are still challenging, since they are rare, and the amount of available samples are very limited. This review will focus on the clinical potential of ct-DNA and CTCs in both the early and advanced diagnosis, prognosis, and in the identification of resistance mutations in OC.

## Background

Ovarian cancer (OC) is the fifth most common cause of cancer death and remains the most leading cause of gynecological death [1]. These poor outcomes are directly related to the fact that a large majority, almost 75% of ovarian cancers, are diagnosed at advanced stage (III/IV), when transperitoneal, hematogeneous, and lymphatic dissemination have already occurred [2]. In general, effective therapy in OC patients can achieve 90% when the tumor is still confined to the ovary; unfortunately, only 25% of OC can be diagnosed before it exacerbates [3]. Despite the modern management, introduction of improved surgical techniques, combination chemotherapy and targeted therapies, the overall survival rates for these patients have not been significantly improved, with 70% of all OC patients succumbing to it within 5 years [4]. Multiple efforts have been made to improve survival rates through early screening methods based on serum cancer antigen 125 (CA-125) concentrations and transvaginal ultrasound [5, 6]; however, these methods have not met the standards we expected, to advocate population-based screening. Although the CA-125 and transvaginal ultrasound are currently the two main techniques used to diagnose OC, they lack both sensitivity and specificity for early detection of OC. Moreover, acquired drug resistance to chemotherapies is ubiquitous during the progression of the disease. These indicate that there is an urgent need for additional cancer-specific diagnostic biomarkers to monitor tumor evolution and predict the onset of resistance to chemotherapies.

Currently, tumor biopsy samples are regularly performed in routine clinical practice with the purpose to evaluate specific biomarkers predicting therapy response [7]. However, it might be inadequate to characterize the genetic heterogeneity of tumor progression with a single biopsy, which does not yield comprehensive information of tumor genome.

Recently, the use of circulating cell-free DNA (cf-DNA) as a biomarker has attracted attention, due to various types of DNA alterations being reported in cf-DNA, including point mutations, microsatellite instabilities, DNA hypermethylations and loses of heterozygosity. These alterations, in many cases, were found to be identical to the ones discovered in the primary tissues from the patient, suggesting cf-DNA may be a valuable source of genetic material as a surrogate for molecular analysis in diagnosis and prognosis.

The circulating tumor cells (CTCs) analysis have also demonstrated predictive and prognostic value for both early and advanced cancer patients. Elevated CTCs in patients during the course of treatment have a significantly shorter, progression-free survival (PFS), as well as overall survival (OS). High heterogeneity has been observed by directly measuring gene expression in individual CTCs [8].

The cf-DNA and CTCs are potential surrogates for the tumor itself, offering favorable potential for serial monitoring of tumor genomes in a noninvasive approach [7]. Therefore, liquid biopsies may be feasible to improve the sensitivity and specificity, promoting earlier detection of recurrences and likelihood of cure, ensuring better diagnostics, treatment decisions and optimal management in OC.

## Circulating cell-free DNA

Large quantities of tumor DNA were detected in the circulation of cf-DNA found in plasma/serum of cancer patients in 1977 [9]. Even though it has been put forward for nearly 40 years, little information is known about the release of cf-DNA in the circulation. Without question there is a percentage of cf-DNA that comes from nucleated cells [10], however, when it comes to its origin and mechanism we can only postulate. It is assumed that a rate of cf-DNA enters the plasma when the cells on the interface between the primary tumor and the circulation undergo lysis. An additional hypothesis is that both the breakdown of cancer cells and the destruction of tumor micrometastases, contribute to a portion of the release to the bloodstream [11]. The proportion of cf-DNA originating from tumor cells is not only determined by the state and size of the tumor [12], but also by clearance, degradation, lymphatic circulation, and other physiological blood processing [12, 13].

The cf-DNA concentration in plasma/serum of healthy subjects normally ranges from 0 to 100 ng per ml of blood, with an average of 30 ng per ml of cf-DNA [14]. In contrast, the cancer patients have concentrations from 0 and to over 1000 ng per ml of blood, with an average of 180 ng per ml of cf-DNA [15]. The size of DNA fragments released from cells in cancer patients varies between 0.18 to 21 kilobases, with variations from sample to sample, in size distribution of DNA fragments [16, 17].

## Diagnosis

The quantitative analysis of cf-DNA has been used as a non-invasive clinical tool for OC. Significantly elevated cf-DNA levels were detected in the serum/plasma of OC patients when compared with those of healthy individuals, and patients with benign ovarian tumors [18]. Due to the lack of suitable data, we do not know if concentrations in the plasma of OC patients correlate with the ovarian tumor size, stage or location.

While observing both prostate cancer and lung cancer, no correlations between plasma DNA concentration, and disease stage and histological subtypes were found [19–22]. There is also no correlation between cf-DNA concentration and age, sex, or body mass index [23]. But, the cf-DNA levels in stage III and stage IV OC, were found to be higher than those of stageIand stageII OC [24].

When quantitative analyses of cf-DNA were applied for the detection of OC, the results were not consistent and not conclusive. In a meta-analysis of 9 studies evaluating the accuracy of cf-DNA for the diagnosis of OC, the author concluded 70% sensitivity and 90% specificity, suggesting cf-DNA had unsatisfactory sensitivity but acceptable specificity, for diagnosis of OC [25]. Therefore, cf-DNA may be used as an adjuvant diagnostic method for OC because current detection methods lack sufficient specificity such as, the detection based on serum cancer antigen 125 (CA-125), human epididymis protein4 (HE-4) and ultrasound imaging. So the potential use of cf-DNA as a combination application tool, along with the various methods used at present to diagnose OC, may be promising. Studies show that there is no correlation between the serum levels of cf-DNA and CA-125 and HE-4; and when two or three of these biomarkers were used as combined detection, specificity was improved [24].

Chromosomal instability is inherent to OC and is also an important hallmark [26]. The tumor-specific patterns of chromosomal instability can also be detected in cf-DNA [27]. In another study, it was shown that the copy number alterations observed in cell-free DNA, match well to those in tumor biopsies of high-grade serous OC patients; and this proposed method of cell-free DNA testing outperforms both serum CA-125, and the risk of malignancy index [28]. This is a promising method when broadly applied to clinical practice for the prediction of OC. An overview of research studies on cf-DNA in OC was shown in Table 1.

**Table 1:** An overview of cf-DNA in ovarian cancer patients

cf-DNA	Year	Sample Type	NO. Patients	Targeted gene	Diagnosis	Prognosis	Response to therapy	Reference
DNA amount	2015	Serum	36	–	√	–	–	[24]
	2014	Plasma	144	Cyclophilin A	–	√	–	[30]
	2010	Plasma	164	GAPDH, beta-actin	–	√	–	[84]
Reversion mutation	2017	Plasma	19	BRCA1, BRCA2, TP53	–	–	√	[85]
	2017	Plasma	30	BRCA1, BRCA2, TP53	–	–	√	[86]
Somatic mutation	2014	Plasma/serum	1	FGFR2-FAM76A	√	–	–	[87]
	2012	Plasma	15	KRAS, PIK3CA, H1047R	√	√	–	[36]
	2008	Plasma	119	p53	√	–	–	[88]
	2005	Plasma/serum	69	TP53	–	√	–	[89]
Aberrant methylation	2017	Serum	114	RUNX3, TFPI2, OPCML	√	–	–	[90]
	2009	Plasma	33	BRCA1, HIC1, PAX5, PGR-PROX, THBS1	√	–	–	[91]
	2005	Plasma	51	RASSF1A	√	√	–	[92]
	2004	Peritoneal fluids	57	Panel genes including: TIMP3, CDH1, APC, ESR1, BRCA1, MYOD1, GSTM3, TITF1	√	√	–	[93]
Chromosomal abnormality	2017	Plasma	57	–	√	–	–	[27]
	2002	Serum	38	–	√	√	–	[94]
	2001	Serum	30	p53	√	√	–	[95]

cf-DNA; circulating cell-free DNA; √, yes; −, not reported.

An aberrant hypermethylation was found in stage IA/B tumors and low malignant potential borderline neoplasms. In contrast, hypermethylation of the gene panel was not observed in cyst tissue, serum or peritoneal fluid DNA, in benign ovarian disease, normal ovarian tissue DNA, or in healthy age-matched women [29]. Therefore, the analysis of tumor-specific hypermethylation in serum is viable to contribute to the detection of OC.

The use of cf-DNA may help OC patients to receive adequate therapy, increased surveillance or controlled management. However, the potential use of cf-DNA as a biomarker for early diagnosis of OC is the most challenging future application of cf-DNA assessment and will have to overcome many obstacles. Most of the recent reports about the use of cf-DNA in OC are limited to the advanced stage or types of OC. There are also technical hurdles to overcome, such as false positive findings, standardization of plasma processing and data analysis [7].

## Response to therapy

Another objective in the exploration of the use of quantification of cf-DNA technology is to follow patients over time, while observing their response to treatment. It was reported that cf-DNA could significantly discriminate between OC patients before and after chemotherapy, demonstrating that quantification of plasma DNA might develop into a new method to assess the efficacy of chemotherapy [18]. The quantification of cf-DNA also correlates with timing of recurrence.

In another study of 144 patients with epithelial OC who were treated with bevacizumab, both PFS and OS were found to be significantly shorter in patients with high levels of cf-DNA in the blood, suggesting that cf-DNA could be of independent prognostic importance in patients treated with bevacizumb [30].

Detection of mutant DNA in the plasma of patients with OC could also be useful in the follow-up of postsurgical assessment. During a study of 27 OC patients, 12 cases were found to have mutations of p53 in the cancer tissue; and in 2 of those 12 cases, identical mutations were detected in the DNA of their preoperative plasma. Mutant DNA was undetected in after surgery follow-up of these two patients with p53 mutations in their plasma; however, in one case, the p53 mutation re-surfaced 16 months after surgery [31], indicating the detection of mutant DNA in cf-DNA might be promising for future monitoring of the treatment efficacy.

## Identification of resistance mutations

In order to avoid continuing inefficacious therapies and prevent unnecessary side-effects, it is pivotal to detect the appearance of resistance to chemotherapy and targeted agents in the duration of all phases of cancer management. Cisplatin, carboplatin and paclitaxel for advanced OC patients have significantly improved PFS and OS.

However, the acquired resistance to these agents has become a challenging problem to the first-line treatment. The molecular processes of epithelial to mesenchymal transition (EMT) is critical in embryonic development [32], and also contributes to cancer and metastasis [33]. In the model of drug resistant ovarian cancers, it has been found that EMT gene signatures correlate with the presence of drug resistance [34], and the gene associated with EMT may play a significant role in cisplatin resistance in OC [35]. Therefore, the detection of genetic alterations in cf-DNA may become a noninvasive approach to monitor response and resistance to treatment in real-time, and potentially guide the targeted therapy [36].

In a study analyzing plasma cf-DNA in OC patients who received the treatment cisplatin, truncating mutation in the retinoblastoma 1 tumor-suppressor (RB1) was observed to abundantly increase [37]. Loss of heterozygosity was also observed in the 13q containing the RB1 gene, when matching the metastasis biopsies obtained after treatment [38]. Intriguingly, the loss with RB1 is associated with EMT and has been linked with chemotherapy response [39].

These solid proofs suggest that exome-wide analysis of circulating cell-free tumor DNA could be an adjunct approach to detect mutations relevant to acquired drug resistance in advanced OC patients.

## Circulating tumor cells

CTCs are derived from clones in primary tumor sites [40] and found extremely rare in healthy subjects [41]. They are detectable in various metastatic carcinomas and have potential to establish metastasis in different anatomical sites. Their levels are also found to correlate with therapeutic response and survival [42]. Evidence shows that EMT assists the disseminated progression of a single carcinoma cell from primary tumor sites, and enables them to adhere and develop into distant metastases [43]. In recent years, CTCs have been considered a “liquid biopsy” for all metastatic tumors. Isolation and analyzation of captured CTCs, has demonstrated to be a promising method for non-invasive diagnosis, prognosis and real-time monitoring of treatment efficacy and drug resistance. An overview of research studies on CTCs in OC was presented in Table 2.

**Table 2:** Detection and prognostic relevance of CTCs in ovarian cancer patients

Year	Sample	Sample timing	NO. Patients	Method of CTCs detection	CTCs Positive Rate	Targeted antigen/gene	Prognostic Significance	Reference
2017	PB	Before surgery	54	Biotin-doped Ppydeposited microfluidic device	98.1%	EpCAM, TROP-2, EGFR, vimentin, and N-cadherin	PFS	[96]
2016	PB	Before surgery	56	Size-dependent seperation	58%	Gene panel including: EpCAM, MUC1, MUC16, CK18, 19, ERCC1	NR	[59]
2014	PB	Before surgery	80	RT-PCR	47.5%	MAGE-As	OS, PFS	[97]
2013	BM	Intra-operative period	456	ICC	27%	A-45B/B3	PFS	[98]
2011	PB	Before 2nd line chemotherapy	216	CellSearch®	14.4%	EpCAM, CK, CD45	OS, PFS	[57]
2011	PB	Before surgery	86	RT-PCR	19%	EpCAM, MUC1, HER2	OS	[99]
2009	PB	Before surgery	71	ICC	60.6%	EpCAM, ESA, CK	PFS	[54]
2009	BM	Intra-operative period	112	ICC	25%	A-45B/B3	PFS	[100]
2008	PM	Before surgery	20	Density gradient/flow cytometry	90%	Folate-AlexaFluor 488 DUPA-FITC	NS	[101]
2007	PB/BM	Before and after chemotherapy.	57(PB)/46(BM)	ICC	21%/54%	A-45B/B3, CK8, 18, 19	OS, PFS	[102]
2006	PB	Before and after surgery	24	Immunomagnetic beads/RT-PCR	75%	BER-EP4	NS	[103]
2003	PB	Before surgery	64	ICC	18.7%	CK7, 8, 18, 20, EGFR	NS	[104]
2002	PB/BM	After surgery and before adjuvant chemotherapy	90(PB)/73(BM)	Immunomagnetic beads	12%//21%	MOC-31	NS	[105]

PB, peripheral blood; BM, bone marrow; NR, not reported; NS, not significance; OS, overall survival; PFS, progression-free survival; RT-PCR, Reverse transcription-polymerase chain reaction; CK, cytokeratin; ICC, immunocytochemistry.

CTCs detection methods include two categories, cell surface marker-dependent and marker independent, approaches. Epithelial cell adhesion molecule (EpCAM) is the most commonly used epithelial cell surface markers but is absent from normal leukocytes [44]. The CellSearch (Veridex) system utilizes ferrofluids loaded with an EpCAM antibody to capture CTCs, staining positive for cytokeratins and negative for CD45 [41], and is currently the only U.S. Food and Drug Administration-approved technology for enumeration and detection of CTCs. However, the levels of CTCs which have hindered detection and molecular characterization [45], are extremely low in circulation with an estimated range from one in 100 million to one in a billion normal blood cells in advanced cancer patients [44, 46]. The use of CellSearch so far has also been limited to certain types of cancer, including metastasis breast cancer [47], colorectal cancer [48], and prostate cancer [49]. While using the CellSearch method, the enumerated CTCs of OC patients do not significantly correlate with clinical characteristics or patient outcomes [50]. By contrast, marker independent approaches of isolating CTCs are not limited by the expression of specific cell surface markers, but are involved in biophysical properties of CTCs, such as size [51], deformability [52], or dielectric susceptibility [53]. Some efforts with remarkable results have been achieved and seem to be promising. However, there continues to be an unmet need of methods to evaluate the role of CTCs as biomarkers in OC.

## Diagnosis

In a group of 118 patients diagnosed with OC, CTCs were successfully isolated in 77 patients (65.2%), capturing cells which may assist proliferation potential in different histology OC subtypes [3]. In another group of 71 patients undergoing evaluation for ovarian malignancy, the role of CTCs counts was explored to assess the disease stage. They found that 43 (60.6%) patients had detectable CTCs, including 0/5 benign patients, 1/10 (10%) early stage, 39/52 (73.1%) late stage, and 3/4 (75%) unstaged patients [54]. Unfortunately, the concentration of CTCs presented in OC are extremely low, 1/10^9^ blood cells or 1/10^6^ nucleated blood cells [2], and CTCs counts are not significantly associated with clinical characteristics or patient outcomes [50]. However, the presence of CTCs at diagnosis seems to be correlated with adverse clinicopathological features, and worse clinical outcome, in OC individuals [55] with elevated CA-125 and HE-4 levels [56]. The increased numbers of CTCs also yield an unfavorable prognosis for PFS and OS in OC patients [57]. Its implementation as a valuable diagnostic tool in OC clinical settings, requires uniform methodology and prospective validation. Growing evidence also hints that monitoring CTC-positive individuals may provide an early diagnosis of OC patients. The CTCs could be detected in 24.5% of the baseline, and 20.4% of the follow-up samples in which two thirds identified overexpression of the cyclophilin C gene (PPIC) [56]. A panel of six genes (CCNE2, DKFZp762E1312, EMP2, MAL2, PPIC and SLC6A8) were over-expressed in cancer cell lines and were absent in healthy women. By using this panel, 44% of the cervical cancer patients, 64% of the endometrial cancer patients, and 19% of the OC patients were identified [58]. Interestingly, another study using the MetaCell approach to detect CTCs through vital fluorescene microscopy-based cytomorphologic evaluation in combination with relative gene expression analysis, confirmed a significant expression difference of the KRT7, WT1, EPCAM, MUC16, MUC1, KRT18 and KRT19 genes [59]. Therefore, these genes could be potential biomarkers in assisting early detection and also be predictive of treatment response in gynecological malignancies. However, it is crucial to develop more specific and sensitive technologies that will contribute to early detection of OC.

## Identification of resistance mutation

CTCs not only correlate with advanced stages of OC patients, but also correlate with treatment responses. One of the most interesting applications of CTCs isolation in OC, is the opportunity to detect and monitor the active mutations related to drug resistance. It is widely known that the classical marker EpCAM, for epithelial cells, was found markedly low with only 8% of baseline and 4% of follow-up in CTCs positive samples [56]; indicating it might not be the preferable choice for the isolation or detection of CTCs [60–62]. In contrast, the PPIC in CTCs positive groups was exceedingly over-expressed in baseline groups with 68%, and in follow-up groups with 69% [56]. PPIC gene is identified as a CTC marker [58] belonging to the cyclopilins that have peptidyl-prolyl cis-trans isomerase (PPIase), and are considered intracellular receptors for immunosuppressive drug cyclosporine A (PPIA) [63]. PPIA has also been shown to have significant potential for reversal of platinum chemoresistance [64] and found to be up-regulated in various human cancers with a strong correlation to malignant transformation in several types of cancers [65]. Research has also confirmed that the presence of PPIC positive CTCs, are more likely to be found in platinum resistance samples of follow-up groups, than in platinum sensitive groups [56].

Additionally, another group of genes correlating to chemoresistance was tested (MRP1–10, MDR1, ERCC1, RRM1, RRM2) [59], found to be a promising target for the therapy of chemoresistant epithelial OC patients.

## The added value of cf-DNA and CTCs in the diagnosis of OC

CA-125 is still the main single marker in the diagnostic test of epithelial ovarian cancer (EOC), with its applications for therapeutic efficacy evaluation and monitoring disease status among OC patients [66, 67]. However, CA-125 also increases in benign tumors and in conditions such as endometriosis, follicular cysts, pregnancy and infection [68]. It has limited capacity for diagnostic purpose and classification of ovarian masses as benign or malignant. Ultimately, the diagnosis of specificity of this method based on CA-125 alone is not ideal.

HE4 has emerged as a new diagnostic biomarker for OC and may become as important as CA-125 in OC diagnosis and monitoring of treatment efficacy. HE4 is an N-glycosylated protein encoded by a gene located in chromosome 20q12–13.1. HE4 was found in benign gynecological diseases such as ovarian cyst, uterine fibroids, endometriosis, endometrial polyps and other OCs, including endometrial and cervical cancer, but the expression levels between gynecological diseases and OC are significantly different [69]. Furthermore, HE4 has shown higher sensitivity and specificity than that of CA-125 [70]. The combined analysis of CA-125 and HE4 improves the diagnostic accuracy of OC [71]. Thus, the measurement of serum HE4 can be used for the differential diagnosis between benign gynecologic diseases and OC [72].

CA-125 together with risk of malignancy index values (RMI) were used to construct receiver-operating characteristics (ROC) curve, as compared with cf-DNA testing. The area under the curve (AUC) was assessed, it exhibited that chromosomal instability in cf-DNA testing improved malignancy detection (AUC 0.89), outweighing serum CA-125 (AUC 0.78) as well as the RMI (AUC 0.81). Values of AUC in cf-DNA testing even further increased for high-grade serous cancers specifically (AUC 0.94). Sensitivity of cf-DNA testing was 2 to 5-fold higher compared to CA-125 and RMI testing, by a specificity criteria of 99.6%, the theoretical threshold required for OC screening [27]. The International Ovarian Tumor Analysis Group, IOTA, has initiated promising research to exploit the integration of cf-DNA testing in their prediction models (amendment to IOTA-5 study, NCT01698632) [73]. Thus, cf-DNA testing may be implemented as an important strategy for the detection of OC, due to the potential of improved specificity in combination with well established methods [74].

The detection of CTCs is often hampered by the large number of white blood cells [75] and heterogeneity of primary tumor [76]. No specific marker is uniformly expressed by all cancer types [77], and EpCAM is not an ideal biomarker for CTCs detection as the variation of its gene expression [78]. It is most desirable to develop more specific and sensitive detection methods that will contribute to early detection of OC.

No correlation was observed between the change in CTCs and CA-125 [50], but some patients were negative in CA-125 expression and positive in CTCs. Therefore, CTCs may provide additional prognostic information independently on CA-125. Furthermore the presence of CTCs is significantly associated with shorter OS and PFS [79]. CTCs have exhibited a prognostic value in many types of cancers and shown to be a better monitoring tool in OC than CA-125 [79]. CTCs may become an increasingly valuable tool for the detection of OC, especially in the early stage.

Tumor biopsies are most often obtained in the process of operation. It is difficult and unsafe to take another tumor sample post operation. The molecular characterization of cf-DNA and CTCs can be detected directly in a noninvasive manner in the plasma/serum of cancer patients. Because there is no requirement for enrichment, cf-DNA is easier to obtain compared with CTCs, and the detected gene panels in cf-DNA can be used to monitor treatment response [80]. In contrast, the analysis of CTCs enable the detection of multiple mutations within single cell, which contributes to the understanding of tumor heterogeneity, disease evolution and clinical management [81]. Thus, the assessment of cf-DNA and CTCs mutational profiling could be a substitute source of tumor tissue, providing a combined transcriptome and genome information [82]. It can contribute a better chemotherapy strategy by comparing the alteration frequencies in primary tumors with the increase mutation burden in transcriptome sequencing [83]. Therefore, detecting multiple changes of gene expression from cf-DNA and CTCs can guide its specific targeted therapy and avoid harmful treatment. However, the application of cf-DNA and CTCs into routine clinic still needs more patient data.

## Conclusions

The constant development and discovery of new molecular biomarkers are first and foremost to advance the treatment of OC patients. The emerging of liquid biopsies may represent a turning point in the OC management and could be a novel approach complementing traditional biopsy sampling. The main advantage of liquid biopsy analysis is based on the unique potential of cf-DNA and CTCs to offer minimally invasive samples in a convenient manner and is easily obtainable at multiple time points over the course of disease progression. Evaluation of cf-DNA and CTCs mutational profiling may provide valuable information to early assessment of treatment efficacy in real-time and may enable identification of predictive targets for early diagnosis in OC individuals. Many patients treated with matched targeted therapies develop drug resistance, therefore, it is critical to detect the molecular changes involved in drug resistance. Further research on the molecular characterization of cf-DNA and CTCs will provide a better understanding of the development of resistance to therapies, avoiding repeat biopsies and establishing more personalized treatment with less cost and fewer side effects for OC patients. Liquid biopsy is currently being integrated into preclinical and clinical trials and holds great promise for future genetic studies by providing an evaluation of genomic evolution associated with treatment response and resistance. The application of cf-DNA and CTCs molecular characterization enhances the prediction of response to targeted therapeutics, the identification of subpopulations/gene signatures of cf-DNA and CTCs allowing for patient diagnosis, therapy stratification, accurate prognosis, and a thorough evaluation of patients for clinical trial eligibility. Even though many details remain to be addressed such as, more effective technologies with lower cost, comparisons with other emerging liquid biomarkers, and standardization of assays in evaluating cf-DNA and CTCs, this emerging field of study may bring hope to early detection and monitoring of drug resistance to OC.

## Abbreviations

BM: Bone marrow
CA-125: Serum cancer antigen 125
cf-DNA: Circulating cell-free DNA
CK: Cytokeratin
CTC: Circulating tumor cell
EMT: Epithelial to mesenchymal transition
EpCAM: Epithelial cell adhesion molecule
HE-4: Human epididymis protein4
ICC: Immunocytochemistry
NR: Not reported
NS: Not significance
OC: Ovarian cancer
OS: Overall survival
PB: Peripheral blood
PFS: Progression-free survival
PPIA: Immunosuppressive drug cyclosporine A
PPIase: Peptidyl-prolyl cis-trans isomerase
PPIC: Cyclophilin C gene
RB1: Retinoblastoma 1 tumor-suppressor
RT-PCR: Reverse transcription-polymerase chain reaction
